# AST/ALT ratio as a predictor of mortality and exacerbations of PM/DM-ILD in 1 year—a retrospective cohort study with 522 cases

**DOI:** 10.1186/s13075-020-02286-w

**Published:** 2020-09-20

**Authors:** Renjiao Li, Wen-Jun Zhu, Faping Wang, Xiaoju Tang, Fengming Luo

**Affiliations:** Department of Respiratory and Critical Care Medicine, West China Hospital, Sichuan University, 37 Guoxue Street, Chengdu, 610041 Sichuan China

**Keywords:** PM/DM, ILD, Mortality, Mechanical ventilation

## Abstract

**Objective:**

To assess the associations between aspartate transaminase/alanine transaminase ratio (DRR) and mortality in patients with polymyositis/dermatomyositis-associated interstitial lung disease (PM/DM-ILD).

**Patients and methods:**

This was a retrospective cohort study, which included 522 patients with PM/DM-ILD whose DRR on admission were tested at West China Hospital of Sichuan University during the period from January 1, 2008, to December 31, 2018. Cox regression models were used to estimate hazard ratios for mortality in four predefined DRR strata (≤ 0.91, 0.91–1.26, 1.26–1.73, and > 1.73), after adjusting for age, sex, DRR stratum, diagnosis, overlap syndrome, hemoglobin, platelet count, white blood cell count, the percentage of neutrophils, neutrophil/lymphocyte ratio, albumin, creatine kinase, uric acid/creatinine ratio, triglycerides, or low-density lipoprotein.

**Results:**

Higher DRR (> 1.73) was an independent predictor of 1-year mortality in multivariate Cox regression analysis (hazard ratio 3.423, 95% CI 1.481–7.911, *p* = .004). Patients with higher DRR more often required the use of mechanical ventilation and readmission for acute exacerbation of PM/DM-ILD at 1-year follow-up.

**Conclusion:**

Higher DRR on admission for PM/DM-ILD patients are associated with increased mortality, risk of mechanical ventilation, and hospitalization in 1-year follow-up. This low-cost, easy-to-obtain, rapidly measured biomarker may be useful in the identification of high-risk PM/DM-ILD patients that could benefit from intensive management.

## Introduction

Polymyositis (PM) and dermatomyositis (DM) are common branches of connective tissue disease characterized by muscle weakness and skeletal muscle inflammation, which gradually involves other organs, especially the lungs [1]. Interstitial lung disease (ILD) is a major complication of PM/DM with a prevalence of 20–78% according to studies [2–4]. ILD is also a negative prognostic factor associated with increased morbidity and mortality in patients with PM/DM [5–7]. An Italian study reported that the risk of mortality in patients with PM/DM-associated ILD (PM/DM-ILD) is 2.3 times to that in patients without ILD [8].

De Ritis ratio (DRR), the ratio of serum level of aspartate transaminase (AST) and alanine transaminase (ALT), was first described by Fernando De Ritis in 1957 [9]. AST and ALT are parts of the most common indicators of liver functions, which increase significantly during hepatocellular damage or death [10]. Over the last decades, DRR was considered as a useful indicator of liver diseases, such as hepatitis and liver cancers [11–13]. In recent years, increased levels of DRR have been shown in vascular disorders, including peripheral arterial occlusive disease [14], acute myocardial infarction [15], and acute ischemic stroke [16], and have been associated with malignant tumors [17, 18]. Despite the above studies, to date, no study has evaluated the role of DRR on admission for PM/DM-ILD on the outcome of exacerbation and the long-term survival.

A number of studies have examined potential risk factors associated with poor survival in patients with ILD [19, 20]; however, little is known regarding the usefulness of biomarkers in PM/DM-ILD. Hence, the aim of this study was to identify this easy-to-obtain biomarker for assessing the disease activity of PM/DM-ILD.

## Methods

This was a retrospective cohort study collecting secondary data from West China Hospital of Sichuan University. This study was approved by the Institutional Review Board of West China Hospital, Sichuan University, and written informed consent from patients was waived, but all analyses were carried out after anonymization of patients’ data.

### Patients

All patients who were diagnosed with PM/DM-ILD and had undergone AST and ALT measurement at West China Hospital during the period from January 1, 2008, to December 31, 2018, were eligible for inclusion in the study. All cases were diagnosed and assessed at the department of rheumatology or respiratory medicine. The diagnosis of PM/DM met the Bohan & Peter Diagnostic Criteria [21] and the diagnosis of clinically amyopathic dermatomyositis (CADM) met the criteria developed by Sontheimer and colleagues [22]. Patients with acute hepatic failure or if they had suffered acute myocardial infarction which needed heart bypass surgery or percutaneous coronary intervention in 3 months were excluded. In addition, we excluded patients with chronic renal failure, especially dialysis patients, that significantly influence serum homeostasis, and patients with strenuous exercise within a week.

### Study design

Patient demographics and laboratory and clinical parameters were collected from the medical record. Patient demographics included sex, age, and comorbidities, with special emphasis on hypertension, diabetes mellitus, and chronic hepatitis B. clinical parameters included ventilation, readmission, and diagnosis. Laboratory parameters included hemoglobin (HB), platelet count, white blood cell count (WBC), the percentage of neutrophils, lymphocytes, percentage of eosinophils (EO%), albumin, AST, ALT, uric acid, creatinine, triglycerides, low-density lipoprotein, and creatine kinase (CK). Blood samples were collected from each patient at the time of admission to the emergency department or patients’ fasting blood was obtained in the ward in the next morning, and all samples were sent to the clinical laboratory at West China Hospital.

Patients were evaluated on admission and were followed up for 1 year by monthly outpatient visits in the first 6 months and quarterly in the last 6 months. Routine clinical and laboratory examinations were included in those visits. Complete blood count, liver enzymes, and muscle enzymes were done every visit, and high-resolution computed tomography was done every 3 months. The recording of mechanical ventilation (MV), noninvasive or invasive, was limited to hospital records of West China Hospital, and the recording of readmission for acute exacerbation of PM/DM-ILD (AEPM/DM-ILD) was limited to records of our hospital and confederate hospitals. Acute exacerbation was defined as worsening of dyspnea within 30 days, new radiographic opacities, the presence of new rash, recurrent muscle weakness, or elevated serum levels of muscle enzymes [23–26]. Survival data were retrieved from the electronic records of any accessible hospital in the southwest of China.

### Study outcomes

The primary end-points were all-cause mortality at 1 year. Secondary outcomes included the need for MV and re-hospitalization for AEPM/DM-ILD in 1 year.

### Statistical analysis

Summary statistics for normally distributed quantitative variables were expressed as means and standard deviations. For non-normally distributed variables, we use median and interquartile range. Categorical data were summarized by ratios and percentages. Comparisons between quantitative variables were analyzed by the Student’s *t*, Mann-Whitney *U* test, and Kruskal-Wallis tests based on variable distribution, and Bonferroni’s multiple comparison tests were used for multiple group comparisons. Categorical variables were analyzed by chi-squared test or Fisher’s exact test. Multicollinearity analysis using Spearman’s correlation test (*r* > 0.5) was performed to identify the collinearity between the variables before further analysis.

For the analysis of the objectives, survival analyses and Cox regression analyses were implemented. In detail, we grouped the patients based on quartile of DRR at admission and used the first quartile as the reference group for all subsequent analyses. The times to death according to the DRR strata was evaluated with Kaplan-Meier survival curves and log-rank tests. We conducted univariate analysis by a univariate Cox regression model to assess the relationship between variables and outcomes. Then, we did a multivariable Cox regression to adjust potential confounders, and all variables with *P* < 0.1 in the univariate analysis were subsequently entered into the model as potential predictors. All statistical analyses were performed with the IBM SPSS Statistical version 23.0 (IBM, Armonk, NY, USA SPSS) and graphs were drawn by Graphpad Prism 5.0 (Graphpad Software, La Jolla, CA, USA). A two-sided *p* value less than 0.05 was considered statistically significant.

## Results

### Patient characteristics

A total of 522 patients were enrolled in the study. This cohort included 167 males (mean age 49.54 ± 12.06 years) and 355 females (mean age 50.09 ± 11.25 years). There were 360 (69%), 47 (9%), and 115 (22%) patients who had DM-ILD, CADM-ILD, and PM-ILD, respectively. The prevalence of diabetes mellitus, hypertension, and chronic hepatitis B in this cohort was 6.9% (*n* = 36), 10.3% (*n* = 54), and 4.6% (*n* = 24), respectively. Women had significantly higher median serum AST/ALT ratio and serum globin level than did men (1.30(1–1.82) vs 1.12(0.77–1.56), *p* < 0.001; 31.7(27.6–35.9) vs 29.8(25.4–33.7), *p* = 0.002). Total bilirubin and direct bilirubin were lower in women in this study. Other clinical characteristics are shown in Table 1. Based on the physician’s clinical experience, CS, CS pulse therapy, CYC, CSA, tacrolimus, MTX, AZA, MMF, IVIG, and other treatments were included in the induction treatment regimen. The results of collinearity were summarized in Additional file 1: Figure S1. There were not any variables in this study that highly correlated as indicated by Spearman’s correlation test (*r* > 0.5).

**Table 1:** Characteristics of all patients

Characteristic	All (*n* = 522)	Male (*n* = 167)	Female (*n* = 355)	*p* value
Age, years		49.54 (12.06)*	50.09 (11.25)*	0.619
Diagnosis				0.085
DM	360 (69)	126 (75.4)	234 (65.9)	
CADM	47 (9)	11 (6.6)	36 (10.1)	
PM	115 (22)	30 (18)	85 (23.9)	
OLS		9 (5.4)	41 (11.5)	**0.026**
Comorbidities				
Diabetes mellitus	36 (6.9)	13 (7.8)	23 (6.5)	0.583
Hypertension	54 (10.3)	19 (11.4)	35 (9.9)	0.595
Chronic hepatitis B	24 (4.6)	9 (5.4)	15 (4.2)	0.554
MDA5+	70 (13.4)	25 (15)	45 (12.7)	0.473
HB		130 (120–146)	121 (109–131)	**< 0.001**
PLT		194 (143–254)	195 (148–262)	0.508
EO%		0.6 (0.1–2.6)	0.7 (0.2–2.4)	0.653
NLR		4.52 (3.09–7.82)	4.25 (2.99–6.84)	0.323
TBIL		9 (6.8–12.3)	8.4 (6.5–11)	**0.042**
DBIL		3.3 (2.5–4.6)	3 (2.4–3.9)	**0.005**
IBIL		5.2 (4.1–7.8)	5.5 (4–6.9)	0.441
TP		65.2 (59.8–70.5)	65.9 (61.4–71.1)	0.147
ALB		34.9 (31.3–38.4)	34.3 (30.7–37.9)	0.272
GLB		29.8 (25.4–33.7)	31.7 (27.6–35.9)	**0.002**
AST/ALT		1.12 (0.77–1.56)	1.30 (1–1.82)	**< 0.001**
UA/CREA		5.3 (3.93–6.51)	5.35 (4.29–7.05)	0.062
TG		1.82 (1.35–2.51)	1.97 (1.45–2.79)	**0.016**
LDL		2.46 (1.81–3.05)	2.29 (1.68–3)	0.221
CK		110 (52–703)	136 (42–1156)	0.943
Readmission within 1 year		36 (21.6)	67 (18.9)	0.472
Need for MV within 1 year		15 (9)	28 (7.9)	0.671
Deaths within 1 year		21 (12.6)	43 (12.1)	0.881

Data are presented as n, n (%) for categorical variables and median (interquartile range) for continuous variables

*: mean (standard deviation)

Bold indicates statistical significance

*DM* dermatomyosis, *CADM* clinically asymptomatic dermatomyosis, *PM* polymyosis, *OLS* overlap syndrome, *MDA5* anti-melanoma differentiation-associated gene 5, *HB* hemoglobin, *PLT* platelet count, *WBC* white blood cell count, *EO%* percentage of eosinophils, *NLR* neutrophil/lymphocyte ratio, *TBIL* total bilirubin, *DBIL* direct bilirubin, *IBIL* indirect bilirubin, *TP* total protein, *ALB* albumin, *GLB* globin, *UA* uric acid, *CREA* creatinine, *TG* triglycerides, *LDL* low-density lipoprotein, *CK* creatine kinase, *MV* mechanical ventilation

### AST/ALT ratio (DRR) and associations with clinically relevant outcomes and mortality rates

**Fig. 1:**
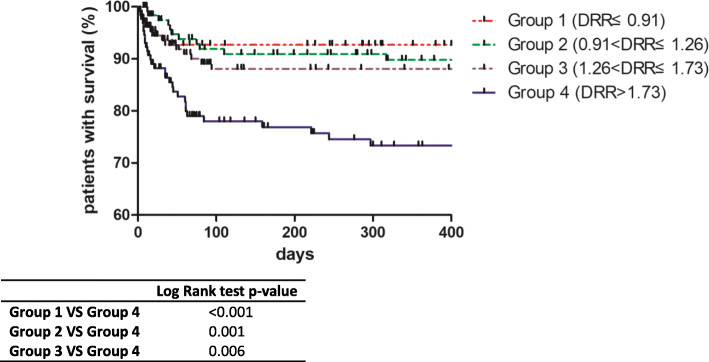
Kaplan-Meier survival analysis across quartiles of AST/ALT ratio

We further divided patients by DRR to explore the relative mortality risk across the DRR strata (Table 2). Cumulative survival rates within 1 year across the DRR strata were 0.927, 0.898, 0.881, and 0.733 (*p* < 0.001). Moreover, patients with higher DRR required MV and readmission within 1 year more often (*p* < 0.05). Figure 1 shows the cumulative survival curve of patients with different DRR strata. Apparently, higher DRR was associated with a poorer prognosis than lower DRR strata (stratum 1 vs stratum 4, *p* < 0.001; stratum 2 vs stratum 4, *p* = 0.001; stratum 3 vs stratum 4, *p* = 0.006), while the prognoses of patients with lower DRR strata did not differ.

**Table 2:** Clinical characteristics of patients across quartiles of AST/ALT ratio

Characteristic	Group 1 (≤ 0.91)	Group 2 (0.91–1.26)	Group 3 (1.26–1.73)	Group 4 (> 1.73)	*p* value
*N*	133	128	131	130	
Follow-up time (days)	365 (53–365)	365 (80.5–365)	365 (67–365)	224 (35–365)	**0.01**
Sex, male	58 (43.6)	38 (29.7)	44 (33.6)	27 (20.8)	**0.001**
Age, years					
≤ 40	31 (23.3)	20 (15.6)	28 (21.4)	22 (16.9)	0.205
40–60	85 (63.9)	85 (66.4)	74 (56.5)	78 (60)	
> 60	17 (12.8)	23 (18)	29 (22.1)	30 (23.1)	
Diagnosis					
DM	82 (61.7)	87 (68)	86 (65.6)	105 (80.8)	**0.046**
CADM	16 (12)	12 (9.4)	12 (9.2)	7 (5.4)	
PM	35 (26.3)	29 (22.7)	33 (25.2)	18 (13.8)	
OLS	11 (8.3)	12 (9.4)	16 (12.2)	11 (8.5)	0.681
Comorbidities					
Diabetes mellitus	8 (6)	11 (8.6)	8 (6.1)	9 (6.9)	0.834
Hypertension	13 (9.8)	20 (15.6)	11 (8.4)	10 (7.7)	0.144
Chronic hepatitis B	6 (4.5)	3 (2.3)	7 (5.3)	8 (6.2)	0.5
MDA5+	18 (13.5)	17 (13.3)	17 (13)	18 (13.8)	0.997
HB	132 (122.5–144.5)	123 (112–132)	120 (111–131)	116.5 (104.5–126)	**< 0.001**
PLT	192 (140.5–233.5)	194.5 (144–262)	195 (154–261)	196 (134.25–264.25)	0.714
WBC	8.07 (6.2–10.63)	6.92 (4.68–10.52)	5.89 (4.49–8.05)	5.41 (3.97–7.81)	**< 0.001**
EO%	0.3 (0–0.9)	0.75 (0.3–2.58)	1.6 (0.3–3.5)	1 (0.18–2.83)	**< 0.001**
NLR	4.28 (3.15–7.72)	4.38 (3.02–6.84)	4.03 (2.99–6.41)	4.82 (2.88–7.93)	0.278
ALB	36.2 (33.55–39.05)	34.75 (31.93–38.48)	34.3 (30.9–38.3)	32.15 (28.3–35.35)	**< 0.001**
UA/CREA	5 (3.98–7.04)	5.24 (4.36–6.58)	5.35 (4.18–6.90)	5.62 (4.32–7.30)	0.476
TG	1.92 (1.37–2.78)	2.16 (1.56–2.79)	1.77 (1.37–2.4)	1.92 (1.44–2.79)	**0.032**
LDL	2.86 (2.3–3.52)	2.44 (1.89–3.11)	2.24 (1.64–2.77)	1.86 (1.32–2.53)	**< 0.001**
CK	69 (30.5–505.5)	106.5 (41–1786.25)	177 (67–1449)	146.5 (59.75–992)	**< 0.001**
Cumulative proportion					
No readmission	0.967	0.908	0.890	0.814	**0.002**
No need for MV	0.944	0.942	0.92	0.805	**0.003**
Survival rate	0.927	0.898	0.881	0.733	**< 0.001**

Data are presented as *n*, *n* (%) for categorical variables and median (interquartile range) for continuous variables

Bold indicates statistical significance

*DM* dermatomyosis, *CADM* clinically asymptomatic dermatomyosis, *PM* polymyosis, *OLS* overlap syndrome, *MDA5* anti-melanoma differentiation-associated gene 5, *HB* hemoglobin, *PLT* platelet count, *WBC* white blood cell count, *EO%* percentage of eosinophils, *NLR* neutrophil/lymphocyte ratio, *ALB* albumin, *UA* uric acid, *CREA* creatinine, *TG* triglycerides, *LDL* low-density lipoprotein, *CK* creatine kinase, *MV* mechanical ventilation

Table 3 shows the unadjusted hazard ratio (HR) for mortality and clinically relevant outcomes across the DRR strata, and Table 4 shows the HRs adjusted by different variables across the DRR strata. In general, the unadjusted HRs for readmission, MV, and all-cause mortality showed a J-shaped association across the DRR strata, which means high DRR stratum was associated with greater risks for readmission, MV, and mortality than low DRR strata. Using the DRR stratum 1 as a reference, the unadjusted HRs (95% CIs) for readmission within 1 year were 1.00, 1.454 (0.794–2.662), 1.346 (0.741–2.446), and 2.664 (1.513–4.692) across the DRR strata. Similarly, unadjusted HRs (95% CIs) for MV within 1 year were 1.00, 0.857 (0.288–2.55), 1.429 (0.544–3.756), and 3.195 (1.35–7.559). Unadjusted HRs (95% CIs) for mortality within 1 year were 1.00, 1.221 (0.506–2.94), 1.554 (0.673–3.59), and 3.668 (1.741–7.729).

**Table 3:** COX univariate analysis

Variables	Readmission within 1 year		Need for MV within 1 year		Deaths within 1 year	
	*p* value	HR	*p* value	HR	*p* value	HR
Sex	0.249		0.530		0.700	
Age, years	**0.077**		**0.042**		**0.017**	
≤ 40						
40–60 (1)						
> 60 (2)						
Diagnosis	**0.028**		**0.035**		**0.093**	
DM						
ADM (1)						
PM (2)						
OLS	0.102		0.148		**0.078**	
Comorbidities						
Diabetes mellitus	0.322		0.395		0.608	
Hypertension	0.353		0.785		0.801	
Chronic hepatitis B	0.233		0.337		0.998	
HB	0.519		**0.062**		0.386	
PLT	0.419		0.598		0.377	
WBC	**0.05**		0.631		0.962	
EO%	**0.017**		**0.001**		**0.017**	
NLR	0.514		**< 0.001**		**< 0.001**	
ALB	0.842		**< 0.001**		**< 0.001**	
UA/CREA	0.669		**0.003**		**0.013**	
AST/ALT	**0.003**		**0.005**		**< 0.001**	
≤ 0.91	Reference		Reference		Reference	
0.91–1.26 (1)	0.225	1.454	0.781	0.857	0.657	1.221
1.26–1.73 (2)	0.329	1.346	0.469	1.429	0.302	1.554
> 1.73 (3)	0.001	2.664	0.008	3.195	0.001	3.668
TG	**0.011**		**0.049**		**< 0.001**	
LDL	0.152		**0.029**		0.149	
CK	**0.096**		**0.082**		**0.086**	

Bold indicates *p* < 0.1

*DM* dermatomyosis, *CADM* clinically asymptomatic dermatomyosis, *PM* polymyosis, *OLS* overlap syndrome, *MDA5* anti-melanoma differentiation-associated gene 5, *HB* hemoglobin, *PLT* platelet count, *WBC* white blood cell count, *EO%* percentage of eosinophils, *NLR* neutrophil/lymphocyte ratio, *ALB* albumin, *UA* uric acid, *CREA* creatinine, *TG* triglycerides, *LDL* low-density lipoprotein, *CK* creatine kinase, *MV* mechanical ventilation

**Table 4:** Cox multivariate regression analysis across quartiles of AST/ALT ratio

AST/ALT ratio	*N* (%)	Unadjusted HR (95%CI)	*p* value	Adjusted HR (95%CI)	*p* value
Mortality*					
≤ 0.91	9 (14.1)	Reference			
0.91–1.26	11 (17.2)	1.221 (0.506–2.947)	0.657	1.259 (0.514–3.086)	0.614
1.26–1.73	14 (21.9)	1.554 (0.673–3.590)	0.302	1.814 (0.757–4.35)	0.182
> 1.73	30 (46.9)	3.668 (1.741–7.729)	**0.001**	3.423 (1.481–7.911)	**0.004**
Mechanical ventilation**					
≤ 0.91	7 (16.3)	Reference			
0.91–1.26	6 (14)	0.857 (0.288–2.550)	0.781	1.086 (0.356–3.312)	0.884
1.26–1.73	10 (23.3)	1.429 (0.544–3.756)	0.469	2.231 (0.791–6.294)	0.129
> 1.73	20 (46.5)	3.195 (1.350–7.559)	**0.008**	4.172 (1.498–11.621)	**0.006**
Readmission***					
≤ 0.91	19 (18.4)	Reference			
0.91–1.26	25 (24.3)	1.454 (0.794–2.662)	0.225	1.62 (0.879–3.066)	0.120
1.26–1.73	26 (25.2)	1.346 (0.741–2.446)	0.329	1.935 (1.020–3.673)	**0.043**
> 1.73	33 (32)	2.664 (1.513–4.692)	**0.001**	3.670 (1.968–6.844)	**< 0.001**

Bold indicates statistical significance

*N* number, *HR* hazard ratio, *CI* confidence interval, *AST* aspartate transaminase, *ALT* alanine transaminase

*HRs were adjusted by sex, age, diagnosis, OLS, EO%, NLR, ALB, UA/CREA, TG, and CK

**HRs were adjusted by sex, age, diagnosis, HB, EO%, NLR, ALB, UA/CREA, TG, LDL, and CK

***HRs were adjusted by sex, age, diagnosis, WBC, EO%, TG, and CK

As shown in Table 4, the adjusted HRs (95% CI) for readmission, MV, and mortality also exhibited a J-shaped association across the DRR strata. The adjusted HRs (95% CI) for readmission were 1.00, 1.830 (0.981–3.412), 2.151 (1.151–4.020), and 4.546 (2.450–8.434); the adjusted HRs (95% CI) for MV were 1.00, 1.086 (0.356–3.312), 2.231 (0.791–6.294), and 4.172 (1.498–11.621); and the adjusted HRs (95% CI) for 1-year mortality were 1.00, 1.259 (0.514–3.086), 1.814 (0.757–4.35), and 3.423 (1.481–7.911).

## Discussion

The present study investigated the association between DRR and mortality. Our analysis demonstrated a significant J-shaped association between DRR and all-cause mortality, with the lowest mortality occurring in individuals with DRR less than 0.91. Additionally, patients with increased DRR more often required the use of MV and readmission for AEPM/DM-ILD at 1-year follow-up. To our knowledge, this is the first study that has assessed DRR, a widely available and rapidly measured biomarker, as a predictor of clinically important outcomes in a retrospective cohort of patients with PM/DM-ILD.

Several mechanisms may be involved in the presence of high DRR in PM/DM-ILD. First, prolonged hypoxemia, caused by exacerbation of diffusing capacity for carbon monoxide (DLco), that is further increased with the development of ILD may result in increased pulmonary artery pressures, leading to increased right ventricle afterload, which promotes hepatocyte injury and AST level increased more [27]. Secondly, PM/DM is characterized by autoimmune conditions that target muscles to some degrees [28], and elevated serum CK, AST, and ALT levels have been associated with increased levels of injury to the skeletal muscle [27, 29]. The positive association of CK with the DRR in our cohort provides support for this mechanism. What is more, pulmonary infection, mainly bacterial, is the most common cause of acute exacerbation in patients with ILD. In the present study, almost all patients who complained of respiratory symptoms, such as cough and dyspnea, were confirmed suffering varying degrees of lung infection in high-resolution computed tomography. Theoretically, serum infectious indicators in these patients, like WBC and neutrophil/lymphocyte ratio (NLR), would increase [30]. However, we found that the difference of WBC between DRR strata was statistically significant: WBC decreased with the increase of DRR, while that of NLR was not, which was a more sensitive marker in infectious diseases. A same trend with WBC was seen in HB as well. There is a possible explanation that patients in higher DRR strata present higher activity of autoimmunity, which attacks blood cells, with a decrease of WBC and HB. More studies are needed to confirm this hypothesis. Finally, ILDs comprise a large group of diseases that generally affect the interstitium [31] and have been associated with increased levels of eosinophils in bronchoalveolar lavage fluid and serum [32, 33], which work as cytokine mediators for acute and chronic inflammation, causing damage to the lung tissues, myocardium, skin, and gastrointestinal tract [34–37]. The positive association of EO% with the DRR in our cohort provides support for this mechanism.

Numerous studies have attempted to identify biomarkers that predict clinically relevant outcomes for PM/DM-ILD. Higher levels of serum Chitinase-3-like-1 protein were negatively associated with DLco and prognoses of PM/DM-ILD in a previous study [38]. In a Japanese study, Yasunori Enomoto et al. showed that higher soluble CD163 levels were associated with worse prognosis and forced volume vital percentage of predicted value [39]. Additionally, the measurement of several serum biomarkers, including Krebs von den Lungen-6 antigen [40], CD4+CXCR4+ T cells [41], microRNA-200c [42], soluble CD206 [43], and progranulin [44], succeeded to predict short-term or long-term prognosis in patients with PM/DM-ILD. The fact that high DDR acted as an independent predictor of 1-year mortality in our study suggests that this easy-to-obtain biomarker may be used to identify high-risk patients that require more intensive treatment.

Previous studies have demonstrated higher DDR was associated with mortality in some malignant diseases, such as renal cell carcinoma, primary hepatic carcinoma, and upper tract urothelial cancer. A retrospective study reported significant associations between serum DRR and renal vein invasion, renal capsule infiltration, and renal pelvis involvement [45]. Another retrospective cohort study in 698 patients receiving nephrectomy has suggested that DRR is related to the prognosis of such patients, but the retrospective design may reduce the generalizability of the application of DRR [17]. And a study of 414 patients with primary hepatic carcinoma in China observed significant associations between DRR and mortality rate [46]. Although all the previously mentioned studies provide evidence that DRR is increased in more severe disease, no study had reported associations between DRR and PM/DM-ILD. In the present study, high DRR was an independent predictor of 1-year mortality in the multivariate Cox regression analysis, which suggests a possible role for this biomarker as a predictor of long-term mortality which needs to be evaluated in larger studies.

Autoimmune features in patients with ILD are important features in the natural history of the disease, and studies have reported that patients with inflammatory myopathies associated ILD have worse morbidity and higher mortality than patients without [47]. A retrospective cohort suggested that poorer pulmonary function test is more common in more severe PM/DM [48] and data from a nationwide prospective Pulmonary Hypertension Registry in France indicated a very possible association between inflammatory myopathies and pulmonary arterial hypertension [49]. This may, in part, explain the associations between high DRR with disease severity and mortality in the 1-year follow-up in our cohort. However, the fact that DRR remained an independent predictor of the risk for hospitalizations in the multivariate Cox regression analysis may suggest a possible role for this biomarker in the identification of exacerbation-prone patients.

In the evaluation of AST and ALT levels, we need to take into account that these enzymes increase in a very sensitive but nonspecific way in several forms of tissue damage and inflammation, especially in muscle and liver tissues, all of which are very dynamic processes in patients with PM/DM-ILD [10]. AST and ALT are influenced by several factors including cardiovascular disease, food intake, exercise, renal dysfunction, and liver diseases. In the present study, we have excluded patients with chronic renal failure and acute hepatic failure, and we excluded patients with strenuous exercise within a week, but we cannot exclude other possible confounders that may have influenced our results. However, despite these possible limitations, we believe that the data from our cohort provide evidence for a possible role of DDR as a biomarker that is associated with disease severity and may identify patients with worse prognosis in hospitalized patients with PM/DM-ILD. A recent study showed that female is associated with poor outcomes in Chinese patients with PM/DM-ILD [50]. The predominance of female patients in stratum 4 of the present study also suggests that the elevated proportion of females may be associated with increased disease-related outcomes in our cohort. However, the fact that higher DDR continued to be an independent predictor of 1-year mortality, even after adjustment for the presence of females, further supports the possible role of DDR as a clinically relevant biomarker in PM/DM-ILD.

The present study presents some limitations. First, as in all retrospective cohort studies, the retrospective nature of data collection could not be avoided. And approximately a third of patients from the analysis were lost to follow-up within 1 year, of which 54 patients lost immediately after their discharge from the hospital. We decided to include them since this was an exploratory noninterventional study evaluating the possible role of DRR as a predictor of clinically relevant outcomes in patients with PM/DM-ILD which we wanted to maintain the integrity of these data and come to the conclusion as accurate as possible. Secondly, for the same reason, we were not able to collect high-quality data on cause-specific mortality. Finally, we do not have sufficient data about the readmission history of AEPM/DM-ILD in our population because of the Two-Way Referral Modes between Hospitals and Community Health Services in China, which restricts access for patients to our hospital, a first-class affiliated hospital. Hence, further studies with prospectively collected data are more appropriate in order to characterize patients as frequent exacerbators in the year of follow-up.

## Conclusion

In conclusion, in the present study, we have shown that the AST/ALT ratio on admission for PM/DM-ILD is associated with increased 1-year mortality, increased risk of mechanical ventilation, and hospitalization for acute exacerbations of PM/DM-ILD in 1 year. Our results, combined with the fact that serum AST and ALT are widely and rapidly available, easy to interpret, low-cost biomarkers, suggest a possible role for AST/ALT ratio in the identification of PM/DM-ILD patients at increased risk of adverse outcomes that may need early intensive management.

## Supplementary information

**Additional file 1.** Multicollinearity analysis using Spearman’s correlation between main variables. HB: hemoglobin, PLT: platelet count, WBC: white blood cell count, NLR: neutrophil/lymphocyte ratio, EO: percentage of eosinophils, AST/ALT: aspartate transaminase/alanine transaminase ratio, ALB: albumin, UA/ CREA: uric acid/creatinine ratio, TG: triglycerides, LDL: low density lipoprotein, CK: creatine kinase.

## Data Availability

The datasets used and analyzed during the current study are available from the corresponding author on reasonable request.

## Abbreviations

PM: Polymyositis
DM: Dermatomyositis
CADM: Clinically amyopathic dermatomyositis
ILD: Interstitial lung disease
PM/DM-ILD: Polymyositis/dermatomyositis-associated interstitial lung disease
PM-ILD: Polymyositis-associated interstitial lung disease
DM-ILD: Dermatomyositis associated interstitial lung disease
CADM-ILD: Interstitial lung disease associated with clinically amyopathic dermatomyositis
DRR: De Ritis ratio
AST: Aspartate transaminase
ALT: Alanine transaminase
HB: Hemoglobin
WBC: White blood cell
EO%: Percentage of eosinophils
CK: Creatine kinase
MV: Mechanical ventilation
AEPM/DM-ILD: Acute exacerbation of PM/DM-ILD
DLco: Diffusing capacity for carbon monoxide
NLR: Neutrophil/lymphocyte ratio
