# Supplementary material for: AST/ALT ratio as a predictor of mortality and exacerbations of PM/DM-ILD in 1 year—a retrospective cohort study with 522 cases
Source: Arthritis Res Ther. 2020 Sep 20;22:202. doi: 10.1186/s13075-020-02286-w (PMC7502203; doi:10.1186/s13075-020-02286-w)
Supplement: Supplementary file 1 — Additional file 1. Multicollinearity analysis using Spearman’s correlation between main variables. HB: hemoglobin, PLT: platelet count, WBC: white blood cell count, NLR: neutrophil/lymphocyte ratio, EO: percentage of eosinophils, AST/ALT: aspartate transaminase/alanine transaminase ratio, ALB: albumin, UA/ CREA: uric acid/creatinine ratio, TG: triglycerides, LDL: low density lipoprotein, CK: creatine kinase. [file 13075_2020_2286_MOESM1_ESM.pdf]

Correlations

			HB	PLT	WBC	NLR	EO	AST/ALT	ALB	UA/CREA	TG	LDL	CK
Spearman's rho	HB	Correlation Coefficient	1.000	-.013	.284**	-.078	.006	-.333**	.454**	.012	.026	.413**	.041
		Sig. (2-tailed)	.	.761	.000	.076	.899	.000	.000	.788	.557	.000	.355
		N	522	522	522	522	522	522	522	522	522	522	522
	PLT	Correlation Coefficient	-.013	1.000	.410**	.139**	.115**	.035	-.084	.113**	-.014	.066	.169**
		Sig. (2-tailed)	.761	.	.000	.001	.008	.429	.055	.010	.747	.134	.000
		N	522	522	522	522	522	522	522	522	522	522	522
	WBC	Correlation Coefficient	.284**	.410**	1.000	.409**	-.114**	-.286**	.055	.048	.033	.268**	.238**
		Sig. (2-tailed)	.000	.000	.	.000	.009	.000	.212	.277	.449	.000	.000
		N	522	522	522	522	522	522	522	522	522	522	522
	NLR	Correlation Coefficient	-.078	.139**	.409**	1.000	-.281**	-.001	-.240**	-.127**	.051	-.015	.027
		Sig. (2-tailed)	.076	.001	.000	.	.000	.985	.000	.004	.247	.734	.533
		N	522	522	522	522	522	522	522	522	522	522	522
	EO	Correlation Coefficient	.006	.115**	-.114**	-.281**	1.000	.180**	.044	.125**	-.072	-.137**	.242**
		Sig. (2-tailed)	.899	.008	.009	.000	.	.000	.319	.004	.101	.002	.000
		N	522	522	522	522	522	522	522	522	522	522	522
	AST/ALT	Correlation Coefficient	-.333**	.035	-.286**	-.001	.180**	1.000	-.315**	.065	.000	-.354**	.185**
		Sig. (2-tailed)	.000	.429	.000	.985	.000	.	.000	.139	.998	.000	.000
		N	522	522	522	522	522	522	522	522	522	522	522
	ALB	Correlation Coefficient	.454**	-.084	.055	-.240**	.044	-.315**	1.000	.029	.075	.368**	-.095*
		Sig. (2-tailed)	.000	.055	.212	.000	.319	.000	.	.515	.088	.000	.030
		N	522	522	522	522	522	522	522	522	522	522	522
	UA/CREA	Correlation Coefficient	.012	.113**	.048	-.127**	.125**	.065	.029	1.000	.087*	.062	.288**
		Sig. (2-tailed)	.788	.010	.277	.004	.004	.139	.515	.	.048	.154	.000
		N	522	522	522	522	522	522	522	522	522	522	522
	TG	Correlation Coefficient	.026	-.014	.033	.051	-.072	.000	.075	.087*	1.000	.115**	.011
		Sig. (2-tailed)	.557	.747	.449	.247	.101	.998	.088	.048	.	.009	.795
		N	522	522	522	522	522	522	522	522	522	522	522
	LDL	Correlation Coefficient	.413**	.066	.268**	-.015	-.137**	-.354**	.368**	.062	.115**	1.000	-.002
		Sig. (2-tailed)	.000	.134	.000	.734	.002	.000	.000	.154	.009	.	.959
		N	522	522	522	522	522	522	522	522	522	522	522
	CK	Correlation Coefficient	.041	.169**	.238**	.027	.242**	.185**	-.095*	.288**	.011	-.002	1.000
		Sig. (2-tailed)	.355	.000	.000	.533	.000	.000	.030	.000	.795	.959	.
		N	522	522	522	522	522	522	522	522	522	522	522

**. Correlation is significant at the 0.01 level (2-tailed).

*. Correlation is significant at the 0.05 level (2-tailed).
